# Phosphorylation of the amyloid precursor protein (APP) at Ser-675 promotes APP processing involving meprin β

**DOI:** 10.1074/jbc.RA119.008310

**Published:** 2019-10-11

**Authors:** Preeti Kumaran Menon, Niina Anneli Koistinen, Kerstin Iverfeldt, Anna-Lena Ström

**Affiliations:** Department of Biochemistry and Biophysics, Stockholm University, 106 91 Stockholm, Sweden

**Keywords:** amyloid precursor protein (APP), amyloid-beta (Aβ), ADAM, Alzheimer's disease, neurodegeneration, β-secretase 1 (BACE1), APP-CTF, meprin β, proteolytic processing

## Abstract

Alzheimer's disease (AD) is a neurodegenerative disorder characterized by abnormal deposition of β-amyloid (Aβ) peptides. Aβ is a cleavage product of the amyloid precursor protein (APP), and aberrant posttranslational modifications of APP can alter APP processing and increase Aβ generation. In the AD brain, seven different residues, including Ser-675 (APP^695^ numbering) in the APP cytoplasmic domain has been found to be phosphorylated. Here, we show that expression of a phosphomimetic variant of Ser-675 in APP (APP-S675E), in human neuroblastoma SK-N-AS cells, reduces secretion of the soluble APP ectodomain (sAPPα), even though the total plasma membrane level of APP was unchanged compared with APP levels in cells expressing APPwt or APP-S675A. Moreover, the level of an alternative larger C-terminal fragment (CTF) increased in the APP-S675E cells, whereas the CTF form that was most abundant in cells expressing APPwt or APP-S675A decreased in the APP-S675E cells. Upon siRNA-mediated knockdown of the astacin metalloprotease meprin β, the levels of the alternative CTF decreased and the CTF ratio was restored back to APPwt levels. Our findings suggest that APP–Ser-675 phosphorylation alters the balance of APP processing, increasing meprin β–mediated and decreasing α-secretase–mediated processing of APP at the plasma membrane. As meprin β cleavage of APP has been shown to result in formation of highly aggregation-prone, truncated Aβ2–40/42 peptides, enhanced APP processing by this enzyme could contribute to AD pathology. We propose that it would be of interest to clarify in future studies how APP–Ser-675 phosphorylation promotes meprin β–mediated APP cleavage.

## Introduction

Alzheimer's disease (AD)^2^ is a neurodegenerative disorder characterized by the formation of senile plaques and neurofibrillary tangles in the brain (reviewed in Ref. 1). β-Amyloid (Aβ) peptides, the main constituent of senile plaques, are generated by sequential cleavage of the amyloid precursor protein (APP) (2). APP is a type I transmembrane glycoprotein with one membrane-spanning domain, a large extracellular N-terminal domain, and a small intracellular C-terminal tail (3). Canonical APP processing can occur through two different pathways, the nonamyloidogenic and the amyloidogenic pathway, in which APP is initially cleaved by α- or β-secretase, respectively, generating either the soluble ectodomain sAPPα or sAPPβ. After α- or β-secretase cleavage, the C-terminal fragments (CTFs) of APP, known as C83 (CTFα) and C99 (CTFβ), respectively, are cleaved by γ-secretase within the transmembrane region, producing either nontoxic p3 peptide and an APP intracellular domain or Aβ species of varying length (Aβ1–40/42) and an APP intracellular domain (4, 5).

The α-secretase cleavage of APP predominantly occurs at the plasma membrane, and two members of the a disintegrin and metalloproteinase (ADAM) family, ADAM10 and ADAM17, have been shown to feature α-secretase activity (4, 5). In contrast, the aspartyl protease BACE1, which is the major Aβ generating β-secretase, is mainly localized to the trans-Golgi network and endosomes, where the acidic environment is optimal for BACE1 activity (4, 5). Recently, evidence that certain amounts of Aβ can also be generated through noncanonical cleavage of APP by meprin β has emerged (reviewed in Ref. 6). Meprin β is a multidomain, type 1 transmembrane, astacin metalloprotease and can cleave APP at the BACE1 cleavage site (P1), but also more prominently at the P2 position, thus generating a CTF (C99*/CTFβ*), one amino acid shorter than C99 (4, 6). Upon cleavage of C99* by γ-secretase, highly aggregate-prone N-terminally truncated Aβ peptides (Aβ2–40/42) are generated (7, 8).

At the plasma membrane, meprin β and ADAM10 may directly compete for APP, as indicated by decreased and increased sAPPα levels in response to meprin β activation and knockout, respectively (8, 9). Moreover, meprin β can activate ADAM10 through cleavage of the pro-peptide (10), and in turn ADAM10 can shed the inactive form of meprin β from the plasma membrane (11). Hence, the balance of ADAM10 *versus* meprin β processing of APP also appears to be regulated by a feedback loop, controlling the activity of these two metalloproteases (for review see Ref. 12).

APP has been shown to undergo extensive posttranslational modifications, including *N*-glycosylation, *O-*glycosylation, ubiquitination, and phosphorylation (13–15). Growing evidence indicates that aberrant posttranslational modifications of APP may play a pivotal role in AD pathogenesis by dysregulating APP processing and promoting Aβ generation. Interestingly, APP contains eight putative phosphorylation sites in the cytoplasmic domain, of which seven (Tyr-653, Ser-655, Thr-668, Ser-675, Tyr-682, Thr-686, and Tyr-687 (APP^695^ numbering)) were recently shown to be phosphorylated in the brains of AD patients (15). Moreover, phosphorylation of several of these sites have been shown to influence APP biology (15–21).

In this study, we characterized the effect of APP–Ser-675 phosphorylation on APP processing and cell surface localization. The study was carried out by mutating APP–Ser-675 to alanine (APP-S675A) or glutamic acid (APP-S675E) to mimic the nonphosphorylated and phosphorylated states of the APP–Ser-675 residue, respectively. The results showed that APP–Ser-675 phosphorylation did not alter the cell surface level of APP. However, the plasma membrane processing of APP was changed, resulting in reduced secretion of sAPPα and total sAPP, whereas increased levels of a C99-like CTF, sensitive to meprin β siRNA knockdown was detected. Taken together these results suggest that phosphorylation of APP–Ser-675 alters the balance between α-secretase and meprin β processing of APP at the cell surface.

## Results

### APP–Ser-675 phosphorylation decreases sAPPα secretion while increasing the level of a slower migrating APP-CTF

**Figure 1.**
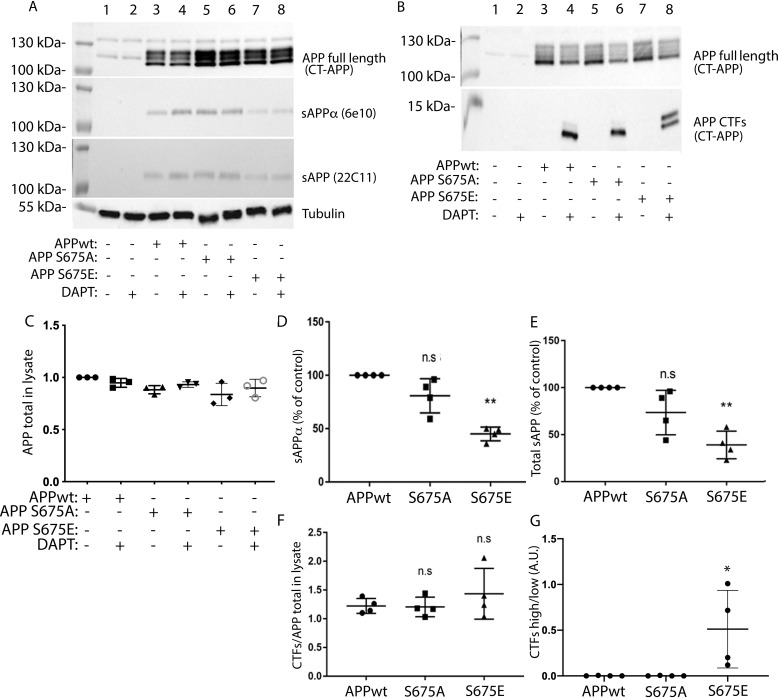
**APP–Ser-675 phosphorylation decreases sAPPα secretion while increasing the level of a slower migrating APP-CTF.** *A*, representative Western blot analysis of full-length APP (detected by CT-APP), sAPPα (detected by 6E10), and total sAPP (detected by 22C11) from nontransfected (*lanes 1* + 2) or APPwt (*lanes 3* + *4*), APP-S675A (*lanes 5* + *6*), or APP-S675E (*lanes 7* + *8*) transfected SK-N-AS cells, in the absence (*lanes 1* + *3* + *5* + *7*) or presence (*lanes 2* + *4* + *6* + *8*) of the γ-secretase inhibitor DAPT. *B*, representative Western blot analysis of APP-CTFs (detected with CT-APP) from nontransfected (*lanes 1* + *2*) or APPwt (*lanes 3* + *4*), APP-S675A (*lanes 5* + *6*), or APP-S675E (*lanes 7* + *8*) transfected SK-N-AS cells, in the absence (*lanes 1* + *3* + *5* + *7*) or presence (*lanes 2* + *4* + *6* + *8*) of the γ-secretase inhibitor DAPT. *C*, quantification of the full-length APP level, normalized against the corresponding tubulin level. *D* and *E*, relative abundance of secreted sAPPα and total sAPP in culture medium from DAPT-treated APPwt, APP-S675A, and APP-S675E overexpressing SK-N-AS cells. The level of sAPPα and total sAPP were normalized against both the level of corresponding total APP expression and the protein concentration in cell lysate. *F*, quantification of the total APP-CTF levels, normalized against the corresponding APP full-length level, in cell lysate from DAPT-treated APPwt, APP-S675A, and APP-S675E overexpressing SK-N-AS cells. *G*, ratio of APP-CTF upper/APP-CTF lower band in cell lysate of DAPT-treated APPwt, APP-S675A, and APP-S675E overexpressing SK-N-AS cells. For quantifications, *, *p* < 0.05; **, *p* < 0.01; *n.s*, not significant; *n* = 3–4.

**Figure 2.**
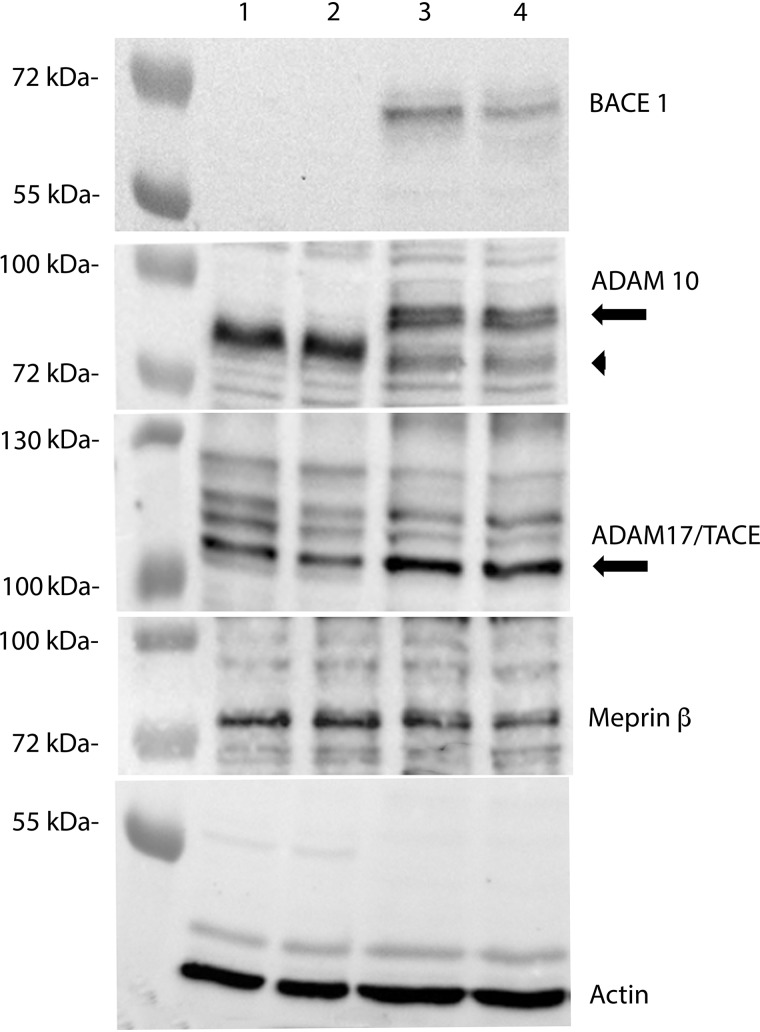
**ADAM10, ADAM17/TACE, and meprin β, but not BACE1, are expressed in SK-N-AS cells.** Representative Western blot analysis of BACE1, ADAM10, ADAM17/TACE, and meprin β expression in two extracts from SK-N-AS (*lane 1* + *2*) and SH-SY5Y (*lane 3* + *4*) cells, respectively. For ADAM10 *arrow* indicates immature ADAM10 and *arrowhead* mature ADAM10.

To investigate the role of APP–Ser-675 phosphorylation on APP processing, a previously described APP^695^-myc cDNA construct (APPwt) (22) was mutated at Ser-675 to either alanine (APP-S675A) or glutamic acid (APP-S675E) to mimic nonphosphorylated and phosphorylated forms of the Ser-675 residue, respectively. Human neuroblastoma SK-N-AS cells were transfected with the different APP constructs and after treatment with the γ-secretase inhibitor DAPT, the full-length APP, sAPPα, and APP-CTF levels were analyzed. A similar expression level of all three full-length APP cDNA constructs was observed (Fig. 1, *A* and *C*). However, although a prominent sAPPα secretion was detected from APPwt and APP-S675A cells, a reduction of sAPPα secretion by ∼50% was observed from APP-S675E cells (Fig. 1, *A* and *D*). Furthermore, although all three APP constructs gave rise to two APP-CTF bands (Fig. 1*B*), a significant difference in the ratio between the two CTFs could be observed in the APP-S675E cells (Fig. 1*G*). The level of the upper, slower migrating CTF band, which was barely detectable in APPwt and APP-S675A cells, was highly increased in cells expressing the APP-S675E phosphomutant (Fig. 1, *B* and *G*). In contrast, the level of the lower, faster migrating CTF was decreased in APP-S675E cells. No significant difference in the total level of CTFs could thus be observed between cells expressing the different APP constructs (Fig. 1*F*). The faster migrating CTF likely corresponds to C83, as a majority of WT APP is normally processed by α-secretase (23). Supporting this, expression of both ADAM10 and ADAM17 α-secretases was confirmed in the SK-N-AS cells (Fig. 2). Taken together, the reduced sAPPα secretion, the increased generation of a slower migrating CTF, and an unchanged total CTF level in the APP-S675E expressing cells suggest that phosphorylation of APP–Ser-675 alters the balance of APP processing, reducing α-secretase mediated cleavage.

**Figure 3.**
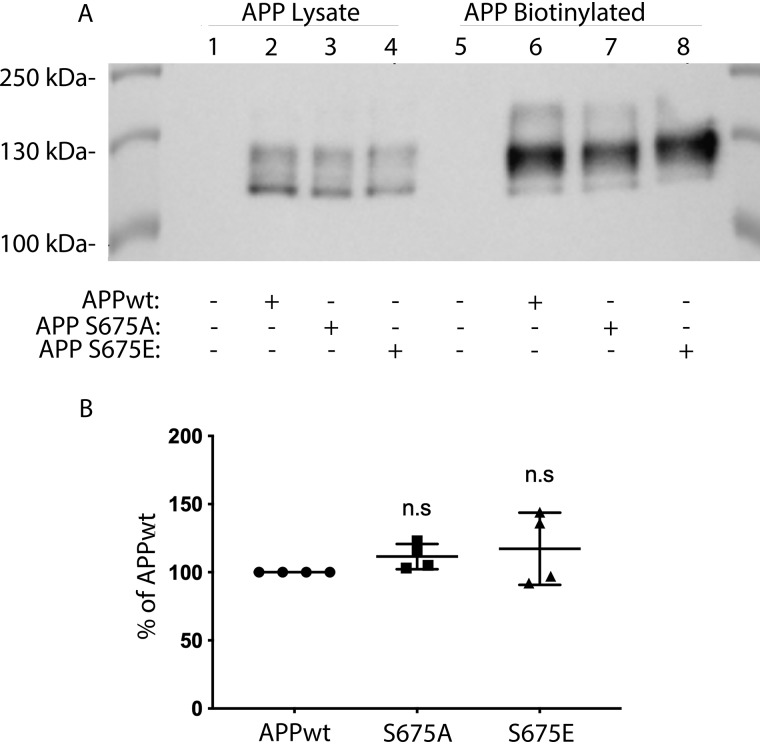
**APP cell surface localization is not affected by APP–Ser-675 phosphorylation.** *A*, representative Western blot analysis of total APP in cell lysate and pulled down biotinylated APP from APPwt (*lanes 2* and *6*), APP-S675A (*lanes 3* and *7*), and APP-S675E (*lanes 4* and *8*) overexpressing SK-N-AS cells. *B*, relative abundance of biotinylated full-length APP from APPwt, APP-S675A, and APP-S675E overexpressing SK-N-AS cells. *n.s*, not significant; *n* = 4.

Because α-secretase processing of APP mainly occurs at the cell surface and reduced APP levels in this compartment could result in reduced sAPPα secretion, we next analyzed the plasma membrane level of APP using a biotinylation assay. However, no significant difference in the total cell surface level of APP could be detected when comparing APP-S675E and APPwt or APP-S675A cells (Fig. 3, *A* and *B*). Hence, the altered processing of APP upon mimicking APP–Ser-675 phosphorylation is not because of altered cell surface localization of APP.

### The slower migrating APP-CTF decreases upon meprin β knockdown

Based on the size, the slower migrating CTF, more abundantly observed in the APP-S675E cells, could correspond to a BACE1-generated C99 or meprin β–generated C99* (4, 5). However, Western blot analysis showed that although meprin β could be detected in SK-N-AS cells, no BACE1 expression could be observed (Fig. 2). This was not because of BACE1 antibody failure, as this secretase could be detected in another cell type (SH-SY5Y) (Fig. 2). Moreover, a shift from α-secretase to more BACE1 processing of APP in the APP-S675E cells should result in an increase of sAPPβ, corresponding to the decrease of sAPPα, thus keeping the total sAPP level detected by the 22C11 APP antibody constant. In contrast, increased meprin β processing of APP has been shown to reduce the level of total sAPP detected by 22C11 (9, 24), possibly because of the three additional meprin β cleavage sites in the ectodomain of APP (24). Analysis of total sAPP secretion from APPwt, APP-S675A, and APP-S675E cells, using the 22C11 antibody, showed that the secretion of total sAPP from APP-S675E cells was reduced to the same extent as the sAPPα secretion (Fig. 1, *A* and *E*). Taken together this suggests that the slower migrating CTF in APP-S675E cells is likely C99* generated by meprin β.

**Figure 4.**
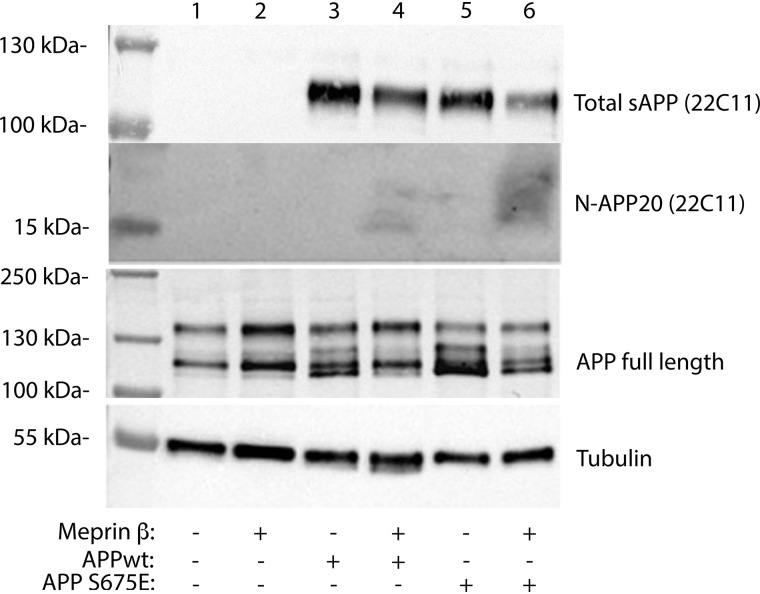
**Analysis of N-terminal APP fragments.** Representative Western blot analysis of total secreted sAPP (detected by 22C11) and N-terminal APP fragments (N-APP20, detected with 22C11) in APPwt (*lanes 3* + *4*), or APP-S675E (*lanes 5* + *6*) expressing SK-N-AS cells, in the absence (*lanes 3* + *5*) or presence (*lanes 4* + *6*) of co-transfected meprin β.

To further test this hypothesis, we next tried to detect the 11 and 20 kDa N-terminal APP fragments, shown to be generated when meprin β cleaves in the APP ectodomain (24). However, no such fragments could be successfully detected in condition media from either APPwt or APP-S675E cells (Fig. 4). In fact, we were unable to clearly detect these N-terminal fragments even if meprin β was overexpressed in the APPwt or APP-S675E cells (Fig. 4). However, a weak smear, more abundant in APP-S675E cells and at the expected size, could be detected upon increasing the meprin β expression. Moreover, a clear reduction of sAPP could be observed upon meprin β overexpression in APPwt cells, similar as in (9). This suggests that even though no N-terminal fragments could be detected, the meprin β overexpression did result in altered APP processing and the N-terminal fragments might be impossible to detect in our experimental setup, perhaps because of rapid degradation.

**Figure 5.**
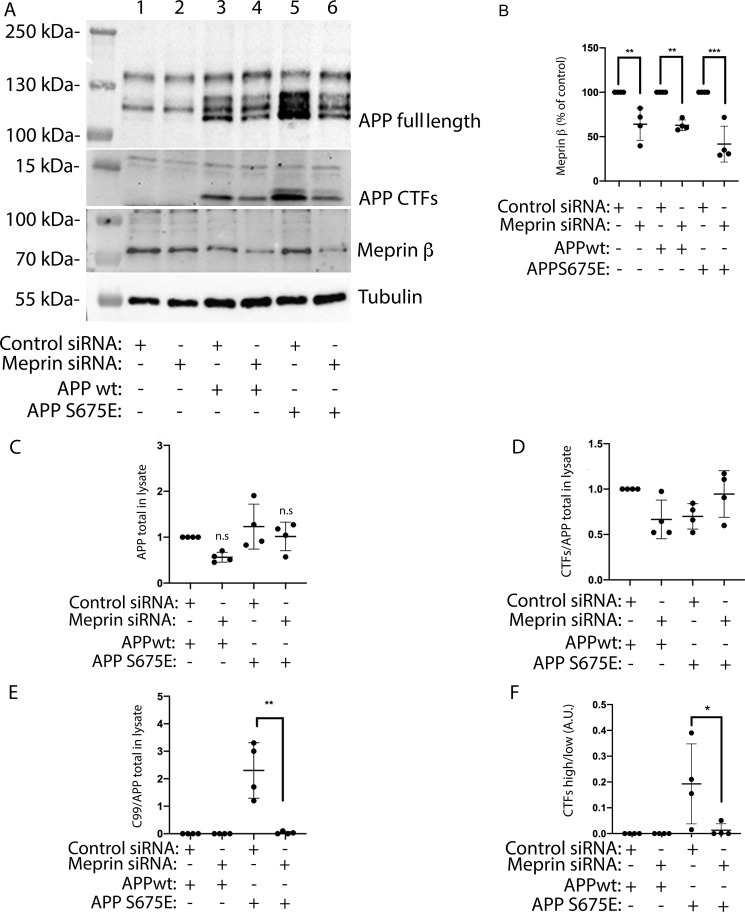
**Meprin β knockdown decreases formation of the slower migrating CTF in APP-S675E cells, without affecting the total level of APP-CTFs.** *A*, representative blot showing the expression of full-length APP, APP-CTFs, and meprin β in DAPT (γ-secretase inhibitor)-treated, non–APP-transfected (*lanes 1* and *2*), APPwt (*lanes 3* and *4*), or APP-S675E (*lanes 5* and 6) overexpressing SK-N-AS cells co-transfected with meprin β (*lanes 2*, *4*, *6*) or control (*lanes 1*, *3*, *5*) siRNA. *B*, quantification of meprin β expression in SK-N-AS cells transfected with control or meprin β siRNA. *C,* quantification of full-length APP, normalized against tubulin, in cells treated as in *A. D*, quantification of the total APP-CTF levels, normalized against the corresponding APP full-length level, in cell lysate from APPwt and APP-S675E overexpressing SK-N-AS cells treated with meprin β or control siRNA. *E*, quantification of the upper slower C99 CTF, normalized against the corresponding APP full-length level, in cell lysate from APPwt and APP-S675E overexpressing SK-N-AS cells treated with meprin β or control siRNA. *F*, ratio of APP-CTF upper/APP-CTF lower band in cell lysate of APPwt and APP-S675E overexpressing SK-N-AS cells, treated with meprin β or control siRNA. For quantifications, *, *p* < 0.05; **, *p* < 0.01; ***, *p* < 0.001; *n* = 4.

To further confirm that meprin β is involved in generating the slower migrating CTF, more abundantly expressed in APP-S675E cells, we next performed knockdown of meprin β in SK-N-AS cells overexpressing APPwt or APP-S675E. Upon meprin β siRNA treatment, which decreased the meprin β expression with ∼40% (Fig. 5, *A* and *B*), a significant decrease in the generation of the slower migrating CTF could be observed in the APP-S675E cells (Fig. 5, *A* and *E*). However, the total level of CTFs did not change (Fig. 5*D*); instead, meprin β knockdown returned the ratio between the upper and lower CTF in the APP-S675E cells back toward the ratio observed in APPwt cells (Fig. 5*F*). These data suggest that meprin β is indeed responsible for the generation of the slower migrating CTF and that phosphorylation of Ser-675 in APP^695^ shifts the balance between α-secretase and meprin β processing of APP, favoring meprin β cleavage.

**Figure 6.**
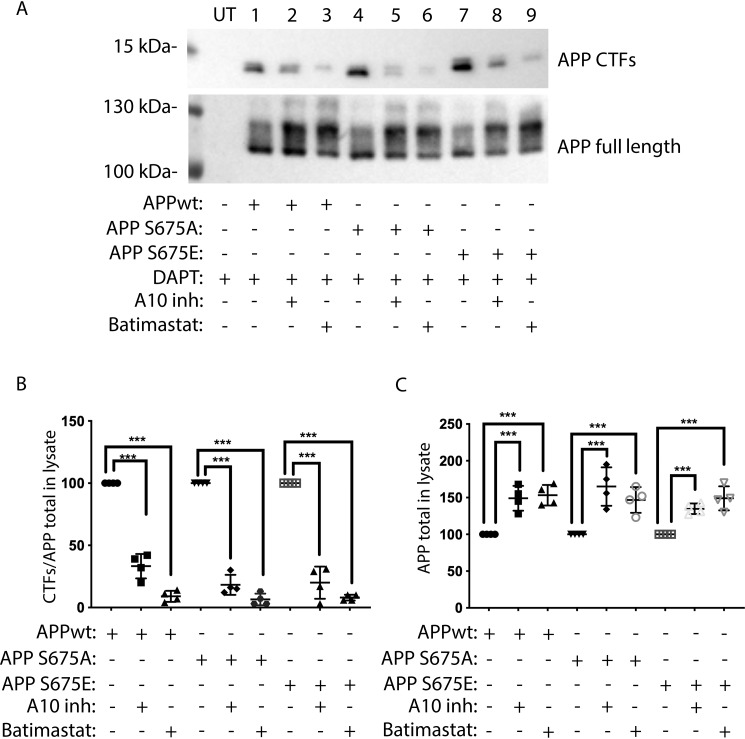
**Metalloproteinase inhibitors decrease the levels of both APP-CTFs generated in APP-S675E expressing cells.** *A*, representative Western blot analysis of full-length APP and APP-CTFs from APPwt (*lanes 1* + *2* + *3*), APP-S675A (*lanes 4* + *5* + *6*), and APP-S675E (*lanes 7* + *8* + *9*) overexpressing SK-N-AS cells, in the presence of the γ-secretase inhibitor DAPT together with either GI254023X (A10inh) or batimastat. *UT*, untransfected. *B*, relative abundance of total APP-CTFs in cell lysate from APPwt, APP-S675A, and APP-S675E overexpressing SK-N-AS cells in the presence of the γ-secretase inhibitor DAPT and either GI254023X (A10inh) or batimastat. *C*, relative abundance of full-length APP in cell lysate from APPwt, APP-S675A, and APP-S675E overexpressing SK-N-AS cells in the presence of the γ-secretase inhibitor DAPT together with either the GI254023X (A10inh) or the batimastat inhibitor. ***, *p* < 0.001; *n* = 4.

To further study the generation of the slower migrating CTF, we next also investigated how metalloproteinase inhibition affected the generation of this APP fragment. SK-N-AS cells overexpressing APPwt, APP-S675A, or APP-S675E were treated with GI254023X (an ADAM10 metalloproteinase selective inhibitor) or batimastat (a broad-spectrum metalloproteinase inhibitor), together with the γ-secretase inhibitor DAPT. Western blot analysis of cell lysates revealed that in the presence of either GI254023X or batimastat, the level of APPwt, APP-S675A, and APP-S675E in cell lysates increased (Fig. 6, *A* and *C*), which is in accordance with the expected accumulation of unprocessed mature APP. As expected, a clear reduction of both the fast and slow migrating CTF could also be observed in all cells following treatment with batimastat, which inhibits both α-secretases and meprin β (25) (Fig. 6, *A* and *B*). However, surprisingly, the ADAM10 selective inhibitor GI254023X did reduced not only the level of the fast migrating CTF, but also the level of the slow migrating CTF, more abundant in APP-S675E cells (Fig. 6, *A* and *B*). This suggests that ADAM10 could be involved in the generation of the slower migrating CTF.

## Discussion

Altered APP processing is believed to play an important role in AD pathology. In this study we for the first time show that phosphorylation of APP–Ser-675, a phosphorylation known to occur in AD brain (15), can regulate the processing of APP. Using APP-S675A and APP-S675E mutants, mimicking the nonphosphorylated and phosphorylated forms of APP–Ser-675, respectively, we found that phosphorylation of Ser-675 significantly reduced the secretion of both sAPPα and sAPPβ without affecting the APP plasma membrane level. Moreover, even in the absence of detectable BACE1 expression, the level of a CTF, similar in size to β-secretase–generated C99, was increased in the APP-S675E cells. In contrast, the level of a smaller CTF, predominantly found in APPwt and APP-S675A cells and, corresponding to C83, was reduced. The total CTF level in APP-S675E cells was thus unaltered compared with in APPwt and APP-S675A cells. This pattern of APP processing, reduced sAPPα, reduced total sAPP, appearance of a C99-like CTF in the absence of BACE1, and a matching decrease of C83, is consistent with what has been observed after increased processing of APP by meprin β (7–9, 24, 26). Meprin β competes with α-secretases and can cleave APP at several sites in the ectodomain, thus precluding production of sAPPα or sAPPβ (7–10, 24). Moreover, cleavage of APP at the BACE1 cleavage site (P1), but also more prominently at the P2 position, leads to the generation of a CTF (C99*/CTFβ*) one amino acid shorter than C99 (7, 8, 26). Subsequent cleavage of this CTF by γ-secretase results in N-terminal truncated Aβ peptides (3, 19, 20). Meprin β processing of APP has been shown to occur *in vivo*, and N-terminal APP ectodomain fragments, as well as N-terminal truncated Aβ fragments, generated by meprin β cleavage, have been detected in the brains of AD patients (8, 10, 24, 27, 28). Moreover, the meprin β cleavage of APP has been shown to be independent of BACE1, as generation of these meprin β APP fragments is not affected by BACE1 inhibitors or knockout (7). In contrast, these APP fragments were abolished in meprin β knockout mice (8, 10). Taken together these studies led to the proposal of meprin β as an alternative β-secretase, for reviews see Refs. 6 and 12). Our results showing generation of a BACE1-independent C99-like CTF, which is reduced by meprin β siRNA, further supports the β-secretase ability of meprin β. Moreover, as we found that the reduction of this C99-like CTF upon meprin β knockdown was accompanied by a corresponding increase in the α-secretase–generated C83 CTF, resulting in no change in overall CTF levels, our data also support the earlier suggestion that α-secretases and meprin β compete for APP at the cell surface (6, 12).

Interestingly, we also found that GI254023X, a selective inhibitor of the α-secretase ADAM10, reduced not only the level of the C83 CTF, but also the C99-like CTF in APP-S675E cells. This raises the question whether ADAM10 could be responsible for the generation of the slower migrating CTF in APP-S675E cells, and the reason meprin β knockdown decreases this CTF might be because of reduced meprin β–mediated activation of ADAM10. However, if this was the case, the meprin β knockdown should also have resulted in reduced ADAM10-mediated generation of C83 and a reduction of the total CTF level in APPwt and APP-S675E cells. This was not the case. ADAM10 have been shown to cleave and thus shed meprin β from the cell surface (11, 29). So instead, could the effect of GI254023X on the C99-like CTF be because of inhibition of ADAM10's ability to shed meprin β? This might seem contradictory as only membrane-bound meprin β can cleave APP at the β-secretase site (7) and reduced shedding upon ADAM10 inhibition could thus be expected to lead to more meprin β–mediated APP processing. However, in the presence of GI254023X, full-length, membrane-bound meprin β was also shown to be released from HEK293T cells via microvesicles (11). This could explain the reduced meprin β cleavage at the APP β-secretase site and the reduced level of the slower migrating CTF in APP-S675E cells upon GI254023X treatment. Moreover, only the inactive form of meprin β was reported to be shed by ADAM10 (11), and how an altered balance of active and inactive meprin β at the plasma membrane affects meprin β–mediated APP processing is not clear. In addition, we cannot completely rule out that the effect of GI254023X on the C99 like CTF is because of a direct inhibitory effect on meprin β, as both ADAM10 and meprin β are phylogenetically related metalloproteases (30). However, the concentration of GI254023X used in this study (2.5 μm) is well below the reported *K_i_* (1.8 × 10^−5^ m) of other ADAM inhibitors like batimastat for meprin β (25). Taken together our results highlight the complex interplay between meprin β and ADAM10, and identify APP-Ser-675 phosphorylation as a novel factor influencing the balance of APP processing by these two proteases.

To our knowledge this is the first time phosphorylation of APP has been shown to alter the balance between canonical and noncanonical APP processing. In contrast, phosphorylation of several other residues in APP, including the most studied, Thr-668, has been suggested to alter the balance between the canonical nonamyloidogenic and amyloidogenic pathways. However, although some studies suggest that APP–Thr-668 phosphorylation elevates BACE1-mediated Aβ generation (15, 31, 32), other studies have found no correlation or attenuated BACE1 cleavage upon Thr-668 phosphorylation (33, 34). Interestingly, a recent study showed that although single Thr-668 or Ser-675 phosphorylation was not, simultaneous phosphorylation of both these residues in APP was sufficient to increase APP internalization and BACE1-mediated Aβ production in response to heighted synaptic activity (17). In accordance with this, we found that Ser-675 phosphorylation by itself did not affect the cell surface level of APP, but rather the processing of APP at the plasma membrane. Hence, based on previous work (17) and our current observations, APP–Ser-675 phosphorylation appears to be an important regulator of APP processing and could promote Aβ production by itself via enhanced meprin β processing or in combination with APP–Thr-668 phosphorylation, resulting in enhanced BACE1 processing.

Although BACE1 is clearly the most quantitatively important enzyme for Aβ generation (35, 36), meprin β could play a role in AD pathology. This idea is supported by the recent identification of a mutation in MEP1B, the gene coding for meprin β, which has a higher frequency in AD cases than controls (37). Moreover, increased levels of both meprin β mRNA and protein have been shown in AD patient brains (7, 26, 38), with especially high expression in neurons and astrocytes in the vicinity of plaques (26). This in combination with the observation that N-terminal truncated Aβ peptides, which can be generated by meprin β but not BACE1 activity, are more aggregation prone and localize to the core of plaques (8, 39) suggests that meprin β could play a role in triggering Aβ pathology. In addition, increased meprin β processing of APP could also contribute to AD pathology by reducing the generation of neuroprotective sAPPα. Further studies to clarify the role of meprin β and how phosphorylation of Ser-675 in APP promotes meprin β processing of this transmembrane protein will thus be of importance.

## Experimental procedures

### Plasmids

The previously described pcDNA3.1 expression vector containing human APP^695^-myc (APP) was used (22). APP-S675A and APP-S675E were generated using the QuikChange II Site-Directed Mutagenesis Kit (Agilent Technologies) according to the manufacturer's protocol. Primer details can be provided on request. The pLBCX-meprin β plasmids were kindly provided by Professor Christoph Becker-Pauly (University of Kiel, Germany).

### Cell culture and treatment

SK-N-AS and SH-SY5Y human neuroblastoma cells (European Collection of Authenticated Cell Cultures) were routinely maintained in minimum essential medium with Earle's salts, 10% fetal bovine serum, 1% l-glutamine, 1% nonessential amino acids, and 1% penicillin streptomycin. All reagents were purchased from Life Technologies. The cells were maintained in a humidified 5% CO_2_ atmosphere at 37 °C.

The SK-N-AS cells were seeded at a density of 25,000 cells/cm^2^ in 60-mm dishes for studies on APP processing upon secretase inhibition and meprin β overexpression. For APP cell surface biotinylation analysis and meprin β siRNA experiments, 100 mm cell cultivation dishes and 6-well plates were used, respectively. 24 h after seeding, SK-N-AS cells were transfected with 2 μg (60-mm dishes) or 5 μg (100-mm dishes) plasmid DNA using X-tremeGENE HP (Roche). Briefly, DNA was mixed with 100 μl (60-mm dishes) or 200 μl (100-mm dishes) MEM and X-tremeGENE HP in a ratio of (μg DNA:μl X-tremeGENE HP) 1:3 for APP and 1:1.5 for meprin β. The mixture was incubated at room temperature for 30 min. For meprin β siRNA knockdown experiments, the cells were transfected with 2 μg (6-well) plasmid DNA and 1 μg (6-well) esiRNA (Sigma Aldrich) for meprin β or *Renilla* Luciferase (negative control) using the X-tremeGENE siRNA transfection reagent according to the manufacturer's instructions.

For studies on APP processing upon α- and γ-secretase inhibition, as well as for meprin β knockdown experiments, media were changed 24 h after transfection and cells then grown for an additional 24 h in the absence or presence of the indicated secretase inhibitors. Secretase inhibitors were used at the following final concentrations; γ-secretase inhibitor DAPT (Sigma) 5 μm, ADAM10 selective inhibitor GI254023X (Sigma) 2.5 μm, and broad-spectrum matrix metalloprotease inhibitor BB-94 batimastat (Sigma) 5 μm.

### Harvesting of cells

Conditioned medium collected from SK-N-AS cells was supplemented with 1× cOmplete Protease Inhibitor mixture (Roche) and then centrifuged to remove cellular debris at 13,000 × *g* for 30 min at 4 °C, before mixing with sample buffer. To analyze the N-terminal fragments from meprin β overexpression studies, 2 ml conditioned medium were concentrated using Amicon ultra-2 centrifugal filter units (3 kDa cutoff, Millipore).

To acquire cell extracts, SK-N-AS and SH-SY5Y cells were washed with 2 × 4 ml ice-cold 1× PBS and lysed for 30 min at 4 °C in RIPA buffer (50 mm Tris-HCl, pH 8, 150 mm NaCl, 1% Nonidet P-40, 0.5% sodium deoxycholate, 0.1% SDS) supplemented with cOmplete Protease Inhibitor. Following centrifugation at 10,000 × *g* for 15 min at 4 °C, supernatants were collected and the total protein concentration in each sample determined using the bicinchoninic (BCA) protein assay kit (Thermo Fisher Scientific).

### Biotinylation assay

SK-N-AS cells expressing APPwt, APP-S675A, or APP-S675E were washed with 2× 10 ml ice-cold PBS supplemented with 1 mm MgCl_2_ and 1 mm CaCl_2_. 5 ml of a 0.25 mg/ml biotin solution (sulfo-NHS-SS Biotin dissolved in PBS supplemented with 1 mm MgCl_2_ and 1 mm CaCl_2_) was then added per plate and the plates incubated for 30 min at 4 °C on a rocking table. The cells were subsequently washed with 3 × 5 ml of Quenching solution (50 mm glycine in PBS supplemented with 1 mm MgCl_2_ and 1 mm CaCl_2_). 150 μl of lysis buffer (10 mm Tris-HCl, pH 7.5, 150 mm NaCl, 0,5% Nonidet P-40, 1 mm MgCl_2_, 1 mm CaCl_2_, + cOmplete Protease Inhibitor) was added to each plate, and cells were lysed for 30 min on a shaker at 4 °C for complete lysis. The plates were scraped and the collected lysates were centrifuged at 13,000 × *g* for 20 min at 4 °C. Dilution buffer (10 mm Tris-HCl, pH 7.5, 150 mm NaCl + cOmplete Protease Inhibitor) was added to the lysate if the final detergent concentration was shown to exceed 0.2% before the total protein concentration of each sample was measured using the BCA protein assay kit. 700 μg of total protein of each sample was added to 200 μl of NeutrAvidine agarose resin (Thermo Scientific) and incubated for 2 h at room temperature on a rocking table. The beads were then centrifuged for 2 min at 5000 × *g* and washed three times with lysis buffer, with mild centrifugation between each washing step. 50 μl of SDS sample buffer was added to the beads, and the beads were boiled for 5 min. Subsequently, the beads were centrifuged for 2 min at 5000 × *g* and the lysates were collected for Western blot analysis.

### Western blot analysis

10 μg of cell lysate for detection of full-length APP, ADAM10, ADAM17, BACE 1, or meprin β, 25 μl of condition medium from each sample for detection of sAPPα and total sAPP and 50 μl of each sample for detection of biotinylated APP, were loaded on 8% Tris-glycine polyacrylamide gels. For detection N-terminal APP fragments, 35 μl of concentrated condition media from each sample was loaded on 4–20% Mini-PROTEAN Tris-glycine precast gels (Bio-Rad, catalogue no. 4561094). 20 μg of cell lysate for detection of APP C-terminal fragments was subjected to electrophoresis on 15% Tris-glycine polyacrylamide gels. Proteins were subsequently transferred to nitrocellulose membrane (GE Healthcare) for 1 h, 500 mA. Nonspecific binding to the membranes was blocked by incubation in 5% nonfat dry milk prior to incubation with a primary antibody overnight at 4 °C, followed by secondary antibody incubation for 30 min at room temperature. Then immunoreactive proteins were detected using the enhanced chemiluminescence (ECL) system (SuperSignal West Dura Chemiluminescent Substrate, Thermo Scientific). Antibody concentrations used were 1:3000 for CT-APP (Sigma), 1:1000 for NT-BACE1 (directed against the N terminus of BACE1, Sigma), 1:1000 for ADAM10 (Millipore), 1:1000 for ADAM17/TACE (Abcam), 1:1000 for 22C11 (directed against 66–81 amino acid residues at N terminus of APP, Invitrogen), 1:1000 for meprin β (R&D Biosystems), 1:1000 for βIII-tubulin (Sigma), 1:1000 for β-actin (Santa Cruz Biotechnology) and 1:2000 for 6E10 (directed against the Aβ sequence, BioLegend). The secondary antibody concentrations were 1:5000 for horseradish peroxidase–coupled anti-goat IgG (Abcam), anti-rabbit IgG (GE Healthcare), and anti-mouse IgG (GE Healthcare).

## Author contributions

P. K. M., N. A. K., K. I., and A.-L. S. conceptualization; P. K. M., N. A. K., K. I., and A.-L. S. formal analysis; P. K. M. and N. A. K. investigation; P. K. M., N. A. K., and A.-L. S. visualization; P. K. M., N. A. K., and K. I. methodology; P. K. M., N. A. K., and A.-L. S. writing-review and editing; N. A. K. and A.-L. S. writing-original draft; K. I. and A.-L. S. supervision; K. I. and A.-L. S. funding acquisition; K. I. and A.-L. S. project administration.

## Footnotes

2 The abbreviations used are:

AD: Alzheimer's disease
Aβ: β-amyloid
ADAM: a disintegrin and metalloprotease
APP: amyloid precursor protein
BACE1: beta-site amyloid precursor protein cleaving enzyme 1
CTF: C-terminal fragment
DAPT: N-[N-(3,5-difluorophenacetyl)-l-alanyl]-*S*-phenylglycine t-butyl ester
sAPP: soluble amyloid precursor protein
TACE: TNF-α converting enzyme.
